# A survey of chloroquine use for prevention and treatment of COVID-19 in Nigeria

**DOI:** 10.4314/ahs.v23i1.10

**Published:** 2023-03

**Authors:** Adeola Yetunde Olukosi, Muinah Fowora, Adeniyi Kazeem Adeneye, Emelda Chukwu, Oluwagbemiga Aina, Olusola Ajibaye, Ayorinde James, Chidinma Gab-Okafor, Susan Abiodun Holdbrooke, Esther Ngozi Ohihoin, Adesola Zaidat Musa, Olufemi Amoo, Oluyomi Showemimo, Bamgboye Afolabi, Oliver Chukwujekwu Ezechi, Babatunde Lawal Salako

**Affiliations:** 1 Biochemistry and Nutrition Department, Nigerian Institute of Medical Research, Yaba, Lagos, Nigeria; 2 Molecular Biology and Biotechnology Department, Nigerian Institute of Medical Research, Yaba, Lagos, Nigeria; 3 Public Health and Epidemiology Department, Nigerian Institute of Medical Research, Yaba, Lagos, Nigeria; 4 Microbiology Department, Nigerian Institute of Medical Research, Yaba, Lagos, Nigeria; 5 Clinical Science Department, Nigerian Institute of Medical Research, Yaba, Lagos, Nigeria; 6 Monitoring and Evaluation Unit, Nigerian Institute of Medical Research Yaba, Lagos. Nigeria; 7 Obafemi Awolowo University, Ile Ife, Osun State, Nigeria; 8 Health, Environment and Development Foundation, Surulere, Lagos State, Nigeria; 9 Nigerian Institute of Medical Research, Yaba, Lagos, Nigeria

**Keywords:** COVID-19, Chloroquine/hydroxychloroquine, self-medication, rational drug use

## Abstract

**Background:**

Rampant chloroquine/hydroxychloroquine poisoning in Nigerian hospitals following suggestions of its possible efficacy in the treatment and prevention of the newly emerged COVID-19 disease informed this survey.

**Objectives:**

The aim of this study was to assess the knowledge, attitude and perception of the Nigerian populace on the use of chloroquine in the COVID-19 pandemic.

**Methods:**

This cross-sectional study was done by administering an electronic questionnaire created using Google Docs, through social media cascade methods including the WhatsApp application software to capture data on chloroquine use between April 20 and June 20, 2020.

**Results:**

Six hundred and twenty-eight people responded to the questionnaire (response rate 99.2%, mean age 41.05 ± 12.3) from the six geopolitical zones in Nigeria with 556 (88.5%) having tertiary level education. Only 21 (3.3%) of the respondents took chloroquine for treatment or prevention. Respondents from the North-west geopolitical zones used chloroquine 5.8 (95% CI: 1.55, 21.52, p=0.02) more times than other zones while the age group 20-29 were 8.8 times more likely to use chloroquine than any other age group (95% CI: 3.53, 21.70, p = 0.00). Female respondents were 2.3 times more likely to use chloroquine than the males (OR 2.26 95% CI: 0.90-5.68; p=0.08) and those in the income bracket of N75,000-99,000, 2.5 times more than other income groups.

**Conclusion:**

Young adults, North-western geopolitical zone, and female gender should be target groups for education on rational chloroquine use. The danger of chloroquine overdose should be communicated to the general population in Nigeria.

## Introduction

The spread of COVID 19 has caused widespread confusion, anxiety, fear, panic, stigma, mistrust, and rumour-mongering. This is especially true because there are no clear-cut solutions to the disease's prevention. To date, no specific pharmacological treatments or chemoprophylaxis for COVID-19 are available. A lot of effort is being put into finding effective drugs against the virus, including repositioning drugs meant to treat other conditions for use as COVID-19 treatment and chemoprophylaxis.^1^,^2^ This list includes chloroquine phosphate and hydroxychloroquine, both of which have undergone clinical trials for the treatment of COVID-19 with varying degrees of success.^2^ It is, however, included in the National Health Commission of the People's Republic of China's sixth version of the COVID-19 treatment guidelines, which established the use of chloroquine nationwide for patients with COVID-19, at a recommended adult dose of 500 mg twice per day for no more than 10 days, but was later revised to a shorter course of 7 days and lower dosage in individuals weighing less than 50Kg.^3^

Malaria, hepatic amoebiasis, lupus erythematosus, light-sensitive skin eruptions, and rheumatoid arthritis are all treated with chloroquine. It functions as an antimalarial, antirheumatic, and dermatologic drug, as well as an autophagy inhibitor.^4^ It was the first-line drug of choice for the treatment of malaria in Nigeria until 2005, when the Malaria Treatment Guideline switched to ACTs due to parasite resistance.^5^ Despite the fact that its use as an antimalarial is prohibited in Nigeria, there are over 20 different brands in the Nigerian market.^6^ As a result, chloroquine is a well-known drug that is inexpensive and easily accessible over the counter. The therapeutic dose for chloroquine-sensitive malaria is approximately 600mg (10 mg base/kg body weight) divided over three days, when as little as 2g can be lethal if not treated immediately. Oral chloroquine is rapidly and nearly completely absorbed, resulting in potentially cardiotoxic transiently high blood concentrations early in the distribution phase. Chloroquine has a large volume of distribution in the human body, with an elimination half-life of 20–60 days and a tendency to accumulate at higher levels in metabolically active tissues than in plasma.^7^,^8^ In retrospective studies, it was discovered that ingestion of 5 g of chloroquine (33 tablets of 155 mg base) by adults was almost always fatal within four hours if no treatment was given.^9^ As a result, with continued use, the recommended dose for COVID-19 treatment can quickly approach danger thresholds. 3 Experts are concerned that false or even dangerous information about what can protect people during a pandemic is spreading at an alarming rate.^10^ For example, after former U.S. President Donald Trump endorsed the anti-malaria drug as a treatment for the novel coronavirus disease, there has been a surge in demand for chloroquine in Nigeria, as well as reported cases of chloroquine poisoning.^11^ This is just one example of a variety of information management scenarios. While many studies have shown that 4-aminoquinolines are active in vitro against a variety of viruses, there is no conclusive evidence of clinical efficacy of chloroquine or hydroxychloroquine, which may be due to the complex pharmacokinetics of 4-aminoquinolines.

Mass media communication has been an important tool in educating the public about misinformation and fake news. The WHO's Risk Communication and Community Engagement (RCCE) Action Plan Guidance of COVID-19 Preparedness and Response are an important component of health emergency preparedness.^12^ A major goal of the RCCE is to proactively communicate and promote a two-way dialogue with communities, the public, and other stakeholders in order to understand risk perceptions, behaviours, and existing barriers, specific needs, knowledge gaps, and provide accurate information tailored to the identified communities/groups.

This survey is an avenue for community engagement centred on chloroquine abuse and the factors that contribute to it. Education and other behavioural change communication deductions will hopefully influence future interventions aimed at COVID-19 at-risk populations and a similar situation. The purpose of this survey was to determine the population's level of knowledge and awareness of the COVID-19 disease, its symptoms, and preventive practices, particularly with regard to chloroquine use.

## Materials and Methods

### Study Design

This study was a cross-sectional online survey assessing the knowledge, attitude, perception and practices of CQ use in the COVID 19 pandemic in Nigeria. Google Docs was used to create a four sectioned electronic questionnaire including A) Socio-demographic characteristics of respondents, B) Knowledge of COVID-19 and Preventive Practices, Section C: Use of preventive medication and Section D: Perception of Chloroquine.

### Study Setting

The data collection tool was administered with the aid of social media communication methods, mainly the WhatsApp networks of faith-based, educational, family and professional group chats. The questions included multiple-choice, text fill, paragraph text, checkboxes and pull-down list. The questions were mostly close-ended, but had options for open-ended answers. Questionnaire was administered between April 20 and June 20, 2020, about when the first positive case was detected in Nigeria. The questionnaire was pilot tested in 25 participants and adjustments were made based on feedback to improve the clarity of the questions. Data of the pilot study were not included in the final analysis. Section-A captured responses to questions on awareness of the presence of COVID-19 in Nigeria, source of information on health issues, awareness and knowledge of its symptoms, management, prevention, risk factors and practices of prevention. Questions eliciting information on knowledge of the use of the drug chloroquine, in particular, source of CQ and sources of advice to use CQ, frequency and dosage of its use, knowledge of who else is using CQ, the brand of chloroquine used, awareness of CQ side effects and dangers of its overdose were also captured by the tool. Data submission was anonymous, so confidentiality issues did not arise.

### Statistical analysis

Descriptive statistics such as frequency (%), mean, and SD was used to present participants characteristics as appropriate. The study questions and characteristics were compared between different categories of participants using independent Student's t-test for continuous variables and the χ2 test for bivariate analysis of categorical data was done using chi-squared and Fisher's exact tests. The level of significance was set at a value < 0.05. Statistical software for social sciences (SPSS) was used for data analysis (ver 26, IBM).

### Ethical issues

The study protocol was reviewed and approved by the Institutional Review Board of the Nigerian Institute of Medical Research; protocol number IRB-20-072. Written informed consent was obtained electronically as a checked “yes” box to participate with the option of a “No” box at which the form was withheld from potential participants.

## Results

### Social Demographics of Respondents

**Figure 1:**
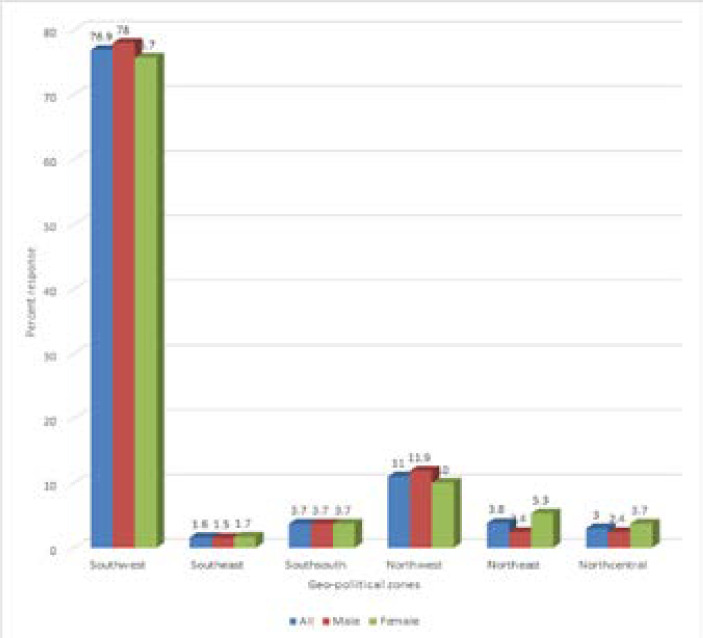
Distribution of response relative to geopolitical zones of the country

A total of 628 (99.2%) individuals consented to participate out of 633 respondents. Table 1 shows the demographic profile of respondents who were mainly from the Southwest zone with 483/628 (76.9%). The participants had a mean age of 41.05 ± 12.3 with the age range 40-49 in the highest proportion 179/628 (28%). Respondents' median monthly income was N50,000 (N0- N and 65.9% of them were married. The majority of the respondents were Christians (79.46%), had attained a tertiary level of education (88.5%) and were in paid employment (52.1%). Almost all of the respondents, 624 (99.4%) were aware of the presence of COVID 19 in Nigeria and a majority (98.1%) believe that it is caused by a virus. Participants from 30 states including the Federal capital territory were represented by the six geopolitical zones in Nigeria with 76.9%, 11.0%, 3.8%, 3.7%, 3.0%, 11.0% and 1.6% of them being from Southwest, Northeast, South-south, Northwest, Northcentral and Southeast geo-political zones respectively as shown in Figure 1.

**Table 1:** Socio-Demographic Characteristics of Respondents

Variable		All (n = 628)		Male (n = 328)		Female (n = 300)		χ^2^ (P-value)
		
		%	Mean (±sd)	%	Mean (±sd)	%	Mean (±sd)	
	All	100.0	41.1 (12.3)	52.2	41.3 (11.8)	47.8	40.8 (12.8)	-
	<20	4.0	18.0 (0.9)	1.8	18.2 (1.0)	4.7	17.9 (0.9)	4.1 (0.04)
	20–29	16.4	24.6 (2.7)	17.7	24.7 (2.7)	16.7	24.4 (2.8)	0.1 (0.74)
Age	30–39	23.7	34.8 (2.7)	23.2	34.8 (2.6)	24.3	34.8 (2.8)	0.1 (0.73)
	40–49	28.0	44.5 (3.0)	30.0	44.6 (3.1)	27.0	44.3 (2.9)	0.3 (0.60)
	50–59	22.6	53.5 (2.7)	24.7	53.6 (2.4)	20.3	53.4 (3.1)	1.7 (0.19)
	≥60	5.3	65.4 (3.5)	3.7	65.3 (4.5)	7.0	65.5 (3.0)	3.5 (0.06)
	None	0.5	-	0.0	-	1.0	-	1.5 (0.22)
	Primary	1.9	-	1.8	-	2.0	-	0.0 (0.88)
Education	Secondary	9.1	-	9.5	-	8.7	-	0.1 (0.73)
	Tertiary	88.5	-	88.7	-	88.3	-	0.0 (0.88)
	Christianity	79.1	-	84.5	-	73.3	-	11.7 (0.0006)
Religion	Islam	18.2	-	13.1	-	23.7	-	11.7 (0.0006)
	Traditional	0.8	-	1.2	-	0.3	-	0.6 (0.42)
	Others	1.9	-	1.2	-	2.7	-	

### Use of Chloroquine for COVID-19 Prevention

Only 21/628 (3.3%) of the respondents had taken chloroquine for prevention against COVID-19 and 15/483(3 .3%) were from Southwest, 3/18 (16.7%) were from the Northwest while 2/18 (11.1%) were from South-south. Chloroquine use was most prevalent amongst those that had tertiary education 20/21(95.2%) and 13(61.9%) of these knew someone else who had taken chloroquine for prevention. When asked how often they took chloroquine, the majority of the respondents who indicated that they took chloroquine stated that they took it daily 7(33.3%) while 6 (28.7%) answered that they took it whenever they remembered. Other responses included; 1 respondent who took it twelve hourly (4.8%), 2 (9.5%) respondents took it once daily, 3 (14.3%) respondents took it weekly and 2 (9.5%) respondents took chloroquine monthly.

**Figure 2:**
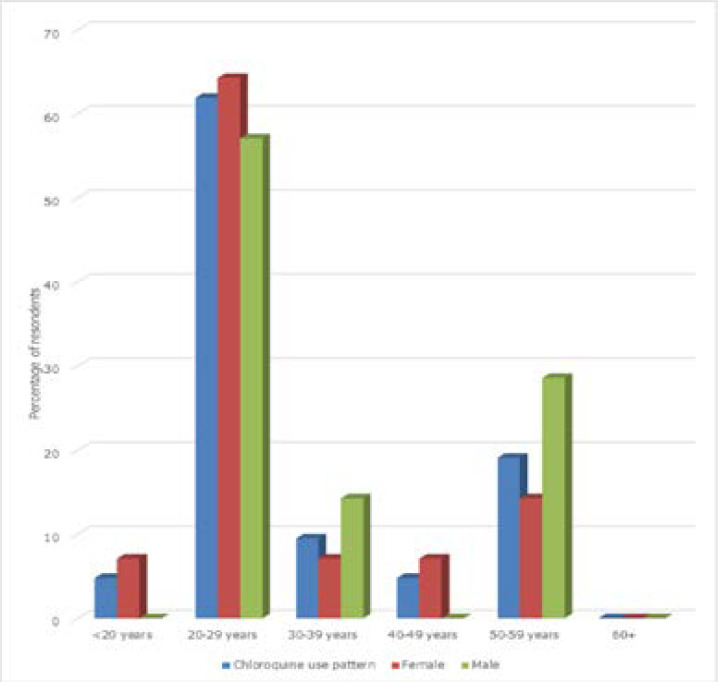
Use of Chloroquine relative to age and gender

Respondents from the Northwest geopolitical zones were more likely to have taken chloroquine for prevention (X2=14.708, p= 0.012). The age range 20 to 29 years used chloroquine the most as represented in Figure 2. The proportion of males and females that used chloroquine for the prevention of COVID-19 was not significantly different but females were 2.3 times more likely to use chloroquine for the prevention of COVID-19 than males (x^2^=3.16, p= 0.08, OR=2.26, 95% CI: 0.90, 5.68). Age group 20-29 were 8.8 times more likely to use CQ than any other age group (OR 8.76, 95% CI: 3.53, 21.70) and those in the income bracket of N75,000-99,000 are about 2.5 times more likely to use chloroquine for the prevention of COVID-19 than any other income group although this difference is not significant (OR 2.35, 95% CI: 0.52, 10.64), Table 2.

**Table 2:** Probability of chloroquine use for COVID 19 prevention relative to socio-demographic factors

Variable	Factor	Age Group					
		
		Yes	No	χ^2^	P-value	OR	95% CI
Age group (years)	< 20	1	19	0.00	1.00	1.55	0.20–12.14
	20–29	13	95	30.45	0.00	8.76	3.53, 21.70
	30–39	2	147	1.68	0.20	0.33	0.08, 1.43
	40–49	1	175	4.70	0.03	0.12	0.02, 0.93
	50–59	4	138	0.02	0.90	0.80	0.27, 2.42
	>=60	0	33	0.36	0.55	-	-
Gender	Male	7	322	3.16	0.08	2.26	0.90, 5.68
	Female	14	285				
Marital status	Single	16	157	26.71	0.0000004	9.17	3.31, 25.49
	Married	4	410	19.15*	0.00001	0.11	0.04, 0.34
	Divorced	1	23	0.00*	1.00	1.27	0.16, 9.87
	Widow(er)	0	17	0.01*	0.93	undefined	Undefined
Income	0	5	105	0.59	0.44	1.49	0.54, 4.17
	1– <25,000	3	122	0.14	0.71	0.66	0.19, 2.29
	25,000–49,000	2	63	0.00	1.00	0.91	0.21, 3.99
	50,000–74,000	2	68	0.00	1.00	0.83	0.19, 3.66
	75,000–99,000	2	26	0.37	0.54	2.35	0.52, 10.64
	≥ 100,000	7	223	0.10	0.75	0.86	0.34, 2.17
Geopolitical Zones	Southwest	16	466	0	1	0.97	0.35, 2.69
	Southeast	0	10	0	1	0	Undefined
	South-south	2	21	0.75	0.39	2.94	0.64, 13.44
	Northcentral	0	69	1.65	0.2	0	Undefined
	Northeast	0	24	0.12	0.73	0	Undefined
	Northwest	3	17	5.36	0.02	5.78	1.55, 21.52

* Fisher's exact

On the other hand, 101 (16.1%) of the respondents had taken orthodox drugs as prevention against COVID 19. There was no significant difference in the use of orthodox drugs across gender, age-range and geopolitical zones. Those that do not believe that the use of chloroquine in the prevention of COVID-19 could be harmful constituted 134 (21.3%) of respondents (Table 1) while 12/628(1.9%) opined that CQ overdose could not cause death. Those that believe that chloroquine can rapidly kill constituted 346/628(55.1%) and those that did not know were 254/628 (40.5%).

### Knowledge of COVID-19

Perceived symptoms, perceived transmission mode, perceived people at risk and perceived side-effects of chloroquine of Covid-19 amongst Nigerians are shown in Figures 3a, 3b, 3c and 3d respectively. The most commonly perceived symptoms of COVID-19 were persistent cough, shortness of breath and fever by 88.9%, 82.8% and 82.3% of respondents respectively. The most commonly perceived transmission mode of COVID-19 was identified as, touching contaminated surfaces, droplets, and 78%, 75%, and 70.4% of respondents respectively while those most perceived as being at risk of COVID-19 are the elderly reported by 84.2%, recent travel to affected region and contact with those who recently travelled to the affected region, as reported by 71.3% and those with chronic diseases by 64.7%.

**Figure 3a:**
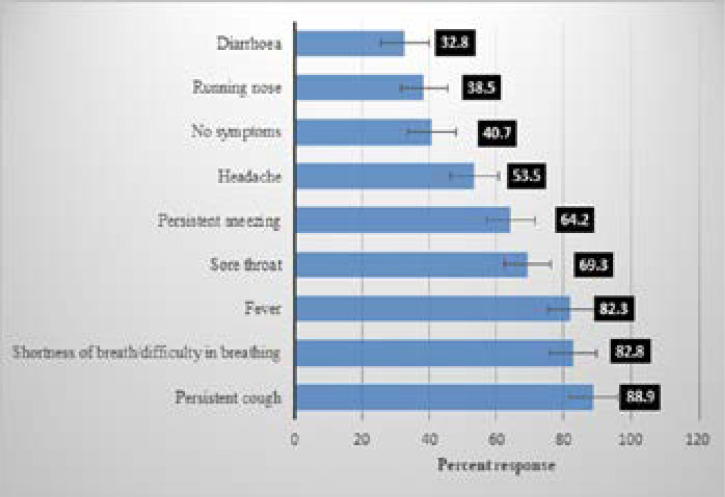
Perceived symptoms of Covid-19 among Nigerians

**Figure 3b:**
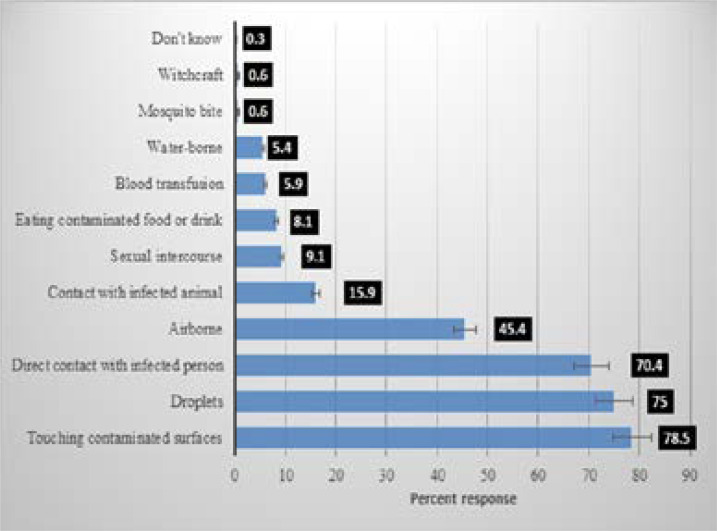
Perceived transmission mode of Covid-19 among Nigerians

**Figure 3c:**
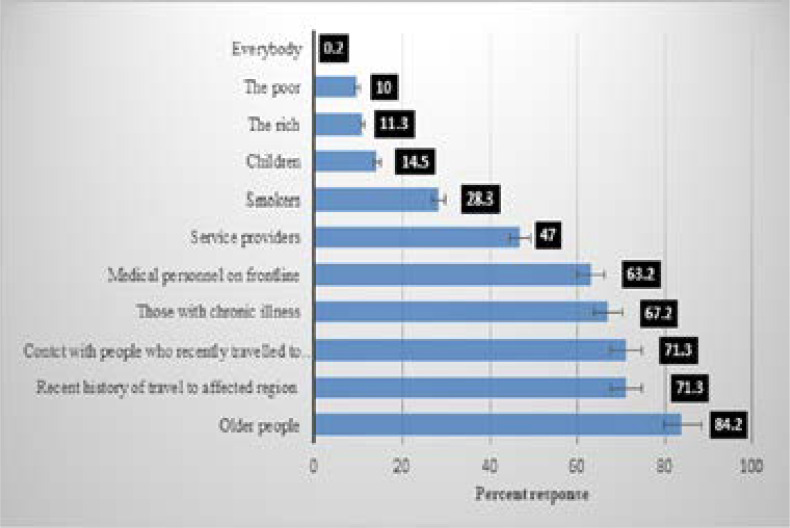
Perceived people at risk of Covid-19 among Nigerians

**Figure 3d:**
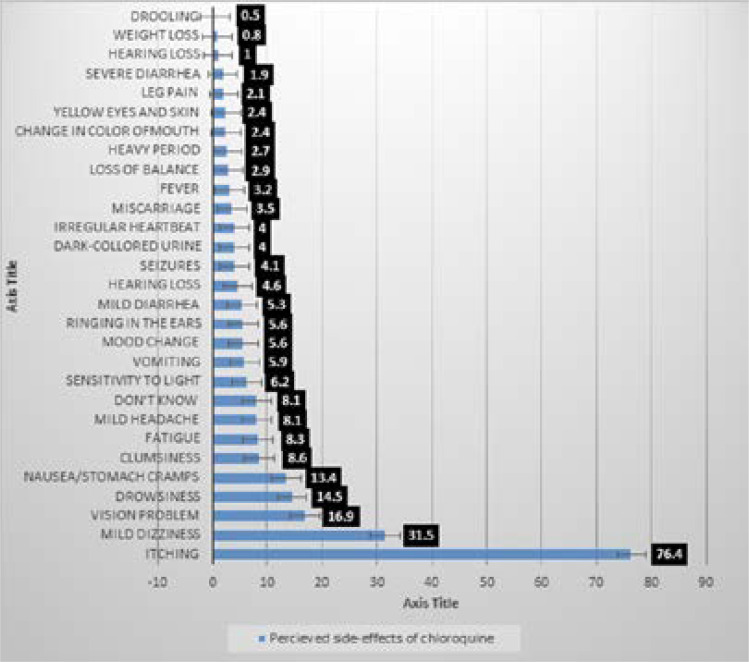
Perceived side-effects of chloroquine

## Discussion

Since the advent of the COVID-19 pandemic, several drugs have been repurposed for the treatment or prevention of SARS-CoV-2 infection.^13^ One of such drugs that has been implicated in the treatment of COVID-19 is chloroquine, leading to a widespread increase in chloroquine demand. This survey was conducted to assess the general public's knowledge, awareness, and perception of COVID-19 and the potential misuse of chloroquine for COVID-19 prevention and treatment in Nigeria.

The awareness and knowledge of COVID-19 were excellent, as almost all of the respondents in our study knew about COVID-19 and its causative agent. The high awareness (99.4%) of COVID-19 reported in this study corroborates that of other studies that assessed the knowledge, attitude, and perception of COVID-19 in Nigeria.^14^,^15^,^16^ These studies also showed that over ninety-nine percent of the studied population had adequate knowledge of COVD-19 and its cause. On the global level, similarly high levels of awareness and knowledge of COVID-19 have been reported in Myanmar, the US, the UK, Italy, Jordan, and China in April 2020, amongst North Americans.^17^,^18^,^19^

Following a world leader's approval of the use of chloroquine for the treatment of COVID-19 symptoms, demand for chloroquine and hydroxychloroquine increased in Nigeria, resulting in a price increase for chloroquine, and some cases of chloroquine poisoning were reported in Lagos State.^20^ Government agencies, including the Nigerian Centre for Disease Control (NCDC) and the National Agency for Food and Drug Administration and Control (NAFDAC), issued warnings as to the trial status of the drug against COVID-19 and warned against the dangers of chloroquine overdose. This, however, may not deter people from abusing chloroquine due to their fears of contracting the virus and their lack of knowledge about the impact of chloroquine overdose. As seen in this study, almost half of the respondents (49%) did not know that the use of chloroquine for prevention could be fatal, but most respondents ticked one or more of the options for chloroquine side effects, with itching (76.4%) and mild dizziness (31.5%) being the two most reported side effects. Nonetheless, the result of the survey demonstrated a low use of chloroquine in the general populace in Nigeria, with only 3.3% of the respondents mentioning taking chloroquine for the prevention of COVID-19. Although the self-reported use of orthodox drugs was not different across the Nigerian geopolitical zones, upon comparing chloroquine use based on a respondent's location, the proportion of those that used chloroquine for COVID-19 prevention was highest in the Northwest geopolitical zone. Interestingly, the majority of the respondents that have used chloroquine for COVID-19 prevention were in the 20–29 age group. This age group was found to be about 9 times more likely to use chloroquine for the prevention of COVID-19 than any other age group. In another study in north central Nigeria, the prevalence of self-medication practices amongst medicine vendors in Jos was highest in the age group of 21-30 years old (86.4%).^21^ A study in Turkey also reported that people in the age range of 18–23 were more likely to practice self-medication.^22^ This further substantiates the increased use of chloroquine self-medication in the 20–29 age group as seen in this study.

## Conclusion

The most at-risk people for using chloroquine for the treatment and prevention of COVID-19 in the Nigerian population surveyed online are young adults, the north-western geopolitical zone, and, to a statistically non-significant extent, the female gender. Educational intervention should be targeted at these groups to discourage non-rational chloroquine use, and the general population should be advised of the danger of overdosing with chloroquine. Assessments will need to be done as the threat of COVID-19 grows, because people's knowledge and beliefs will change. This way, interventions will be relevant to people who need them.
